# Pathogenesis of *Listeria*-Infected *Drosophila wntD* Mutants Is Associated with Elevated Levels of the Novel Immunity Gene *edin*

**DOI:** 10.1371/journal.ppat.1000111

**Published:** 2008-07-25

**Authors:** Michael D. Gordon, Janelle S. Ayres, David S. Schneider, Roel Nusse

**Affiliations:** 1 Department of Developmental Biology, Howard Hughes Medical Institute, Beckman Center, Stanford University School of Medicine, Stanford, California, United States of America; 2 Department of Microbiology and Immunology, Stanford University School of Medicine, Stanford, California, United States of America; Massachusetts General Hospital, United States of America

## Abstract

*Drosophila melanogaster* mount an effective innate immune response against invading microorganisms, but can eventually succumb to persistent pathogenic infections. Understanding of this pathogenesis is limited, but it appears that host factors, induced by microbes, can have a direct cost to the host organism. Mutations in *wntD* cause susceptibility to *Listeria monocytogenes* infection, apparently through the derepression of Toll-Dorsal target genes, some of which are deleterious to survival. Here, we use gene expression profiling to identify genes that may mediate the observed susceptibility of *wntD* mutants to lethal infection. These genes include the TNF family member *eiger* and the novel immunity gene *edin* (*elevated during infection*; synonym *CG32185*), both of which are more strongly induced by infection of *wntD* mutants compared to controls. *edin* is also expressed more highly during infection of wild-type flies with wild-type *Salmonella typhimurium* than with a less pathogenic mutant strain, and its expression is regulated in part by the Imd pathway. Furthermore, overexpression of *edin* can induce age-dependent lethality, while loss of function in *edin* renders flies more susceptible to *Listeria* infection. These results are consistent with a model in which the regulation of host factors, including *edin*, must be tightly controlled to avoid the detrimental consequences of having too much or too little activity.

## Author Summary

Like any organism, fruit flies respond to invading microorganisms by mounting an immune defense. Many aspects of the immune defense in fruit flies are similar to the inflammatory response in mammals, including the harmful effects of a sustained response against persistent pathogenic infections. We found in the past that mutations in the gene *wntD* cause flies to succumb more easily to *Listeria monocytogenes* infections, apparently by losing an element of control over the inflammatory response. How does the *wntD* gene work? In this paper, we have identified genes that may mediate the susceptibility of *wntD* mutants to lethal infection. These genes include *eiger*, a homolog of the mammalian TNF gene, and a previously uncharacterized gene called *edin* (*elevated during infection*). *Edin* is expressed excessively in *wntD* mutant flies, and its expression also correlates with the level of pathogenesis induced by two different strains of *Salmonella typhimurium*. In its own right, overexpression of the *edin* gene can induce lethality, while losing *edin* function renders flies more susceptible to *Listeria* infection. Our results support a model in which the regulation of host factors, including *edin*, must be tightly controlled to avoid the detrimental consequences of having too much or too little activity.

## Introduction

*Drosophila* has an effective innate immune system to combat infection. This response relies heavily on the Toll and Immune deficiency (Imd) pathways, both of which utilize NF-κB related transcription factors as central mediators of signaling: Dorsal and Dorsal-related immunity factor (Dif) in the case of Toll, and Relish (Rel) in the case of Imd (reviewed in [1]–[3]).

The Toll and Imd pathways have largely been characterized with respect to their role in the humoral immune response, a branch of immunity that is triggered through recognition of microbial molecular signatures by upstream components of both the Imd and Toll pathways and subsequent nuclear translocation and activation of the cognate NF-κB factor(s). The activation of these transcription factors leads to transcription of hundreds of genes following infection [4]–[6]. The most studied are the antimicrobial peptide (AMP) genes, which are transcribed in the fat body, leading to secretion of these peptides into the circulating hemolymph (reviewed in [7]).

In addition to its role in AMP regulation, the Toll pathway is also known to participate in two other branches of immunity: the deposition of melanin and the cellular immune response [8]–[12]. The cellular response in particular has become of increasing interest, as studies of *Drosophila* immunity progress beyond the characterization of acute responses to non-pathogenic bacteria to those involving chronic infections that eventually kill the fly [13]–[16]. Many of these model infections proceed intracellularly within the phagocytic cells of the circulating hemolymph, thereby shielding the bacteria from the action of circulating AMPs. This provides a convenient model system for studying the molecular interactions between pathogens and their hosts, including the processes that eventually lead to the host's demise.

One principle that has been understood in mammals for decades, and seems to also be true in *Drosophila*, is that an immune response can be both beneficial and detrimental to a host. Indeed, the same signals that are critical to containing a localized infection will kill the host if uncontrolled [17]. One such signal is Tumor Necrosis Factor (TNF), which is both necessary to fight local infections of many organisms and sufficient to induce lethal septic shock if released systemically [18],[19]. Homologous processes may also occur in *Drosophila*; loss of function mutations in the TNF family member *eiger* result in prolonged survival during infection with *Salmonella typhimurium* [14],[20]. Thus *Drosophila* offers an appealing genetic system to uncover host genes that may have dual effects during the immune response, mediating deleterious consequences to both the pathogen and the host itself.

Previously, we reported evidence that flies mutant for the Wnt family member *wntD* have a defective immune system and succumb prematurely to infection with the gram-positive, lethal bacteria *Listeria monocytogenes* [21]. Given that WntD acts as a feedback inhibitor of Toll-Dorsal signaling during embryonic development [21],[22], we presented a model in which *wntD* mutants exhibit a hyperactivated immune system, including the overexpression of specific Dorsal target genes that are deleterious to the flies' health. Here, we extend those observations by using Affymetrix oligonucleotide arrays to examine the whole genome transcriptional profiles of *wntD* mutants prior to and following infection with *L. monocytogenes*. We examine two groups of candidate mediators of the decreased survival of *wntD* mutants, and provide evidence that one of those genes, *edin* (elevated during infection; synonym CG32185), could be a novel effecter of pathogenesis.

## Results

### *wntD* mutants exhibit upregulation of specific immune targets in the absence of infection

In order to gain insight into the processes that are misregulated in *wntD* mutants and that may contribute to their susceptibility to *L. monocytogenes* infection, we collected RNA from *wntD* and control flies under two conditions: naïve and 24 hours following infection with *L. monocytogenes*. This time point was chosen because we had observed significant mortality of *wntD* mutants between 24 and 48 hours under these infection conditions, and hypothesized that misregulation of genes causally involved in this mortality would be seen most clearly at the beginning of this time window [21].

**Figure 1:**
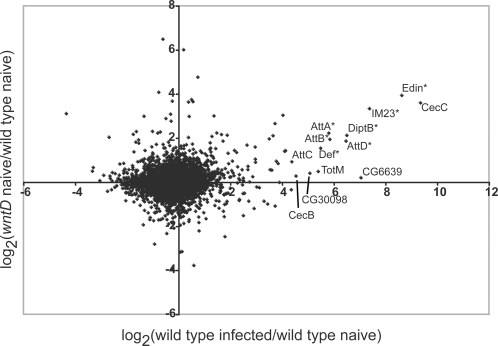
Genes elevated in *wntD* mutants correlate with those elevated by infection. Scatter plot illustrates correlation between Affymetrix gene expression data for log_2_(*yw* infected with *L. monocytogenes*/*yw* uninfected) and log_2_(*yw; wntD* ^KO1^ uninfected/*yw* uninfected). Each ratio described above was the average across 3 samples for each condition. Correlation coefficient, r = 0.14. N = 12047. Significance of correlation calculated as p<0.0001 using the equation t = r/sqrt[(1-r^2^)/(N-2)], with N-2 degrees of freedom. The identity of top 13 genes most elevated by infection are shown. Asterisks indicate genes significantly elevated in *wntD* mutants versus controls (p<0.025).

Previously, we showed that *wntD* mutants exhibit elevated expression of the AMP *Diptericin* prior to and following infection with the non-pathogenic bacterium *Micrococcus luteus*, while the AMP *Drosomycin* is expressed in *wntD* mutants at levels indistinguishable from wild type [21]. To test the idea that *wntD* mutants have a hyper activated basal immune system on a more global scale, we used our array data to look at the correlation between each gene's response to infection in wild type (log_2_(infected controls/uninfected controls)) and its level of misregulation in *wntD* mutants prior to infection (log_2_(uninfected *wntD*/uninfected controls)). As shown in Figure 1, the top thirteen genes most induced by infection all showed higher levels of expression in uninfected *wntD* mutants compared to uninfected controls. Of these thirteen genes, seven showed an average of greater than 2-fold difference between mutants and controls and had p-values less than 0.025 (Figure 1 and Table 1). This set of genes was comprised of the novel immunity gene *edin*, *IM23*, *AttD*, *AttB*, *AttA*, *DiptB*, *and Def*, all of which are known to be induced by infection under various conditions [5],[23],[24]. It is worthwhile noting, however, that several known immune-regulated genes that were strongly induced by infection in our study showed no significant difference between *wntD* mutants and controls, including *CG6639*, *CecB*, *TotM* and *Dros* (Figure 1 and data not shown).

**Table 1:** List of top 13 genes most induced by infection of wild-type flies.

Gene	WT infected/WT uninfected	t-test	*wntD* uninfected/WT uninfected	t-test	*wntD* infected/WT infected	t-test
*Cecropin C*	652.54	0.003	12.16	0.2	0.70	0.01
*edin*	394.73	0.008	15.36	0.02	5.14	0.00001
*IM23*	166.01	0.04	10.14	0.0009	0.98	0.9
*CG6639*	131.72	0.002	1.15	0.8	0.21	0.0002
*Diptericin B*	90.56	0.005	4.36	0.02	1.04	0.6
*Attacin D*	88.19	0.0004	3.65	0.008	1.87	0.004
*Attacin B*	57.37	0.0009	3.85	0.003	1.04	0.3
*Attacin A*	55.71	0.007	4.70	0.02	1.67	0.004
*Defensin*	44.85	0.001	2.92	0.008	1.30	0.01
*Turandot M*	41.93	0.03	1.40	0.5	1.68	0.04
*CG30098*	33.55	0.01	1.33	0.08	1.55	0.2
*Cecropin B*	23.08	0.01	1.21	0.2	0.97	0.8
*Attacin C*	20.70	0.008	1.90	0.09	1.09	0.4

“WT infected/WT uninfected” shows the induction of each gene by infection of wild-type flies with *L. monocytogenes*. “*wntD* uninfected/WT uninfected” shows the enrichment of each gene in *wntD* mutants prior to infection. “*wntD* infected/WT infected” shows the enrichment of each gene in *wntD* mutants following infection. t-test columns indicate the p-value for the comparison given in the leftward column.

Overall, the correlation coefficient (r) for these data sets was 0.14, with a p-value<0.0001. Calculating the coefficient of determination (r^2^) suggests that approximately 2% of the variation within the data can be explained by the correlation between the two data sets. This corresponds to approximately 235 genes, a plausible number given previous studies have indicated that about 400 genes are significantly regulated by infection [4]. In a similar analysis looking at the misregulation of immune genes in *wntD* mutants following infection, no significant correlation was observed (data not shown). As is evident from the cluster analysis presented below and the data in Table 1, a subset of immune-induced genes were expressed more highly in *wntD* mutants following infection, but many of the most highly induced immunity genes were not significantly different between *wntD* mutants and controls, and some were expressed at lower levels in the mutants. This may have resulted from a lack of sensitivity from the array at these high levels of expression, saturation of the signaling processes leading to induction of expression, or dominant negative effects of activated Dorsal on the activity of other NF-κB proteins.

### Cluster analysis reveals two groups of candidate mediators of *wntD* lethality

**Figure 2:**
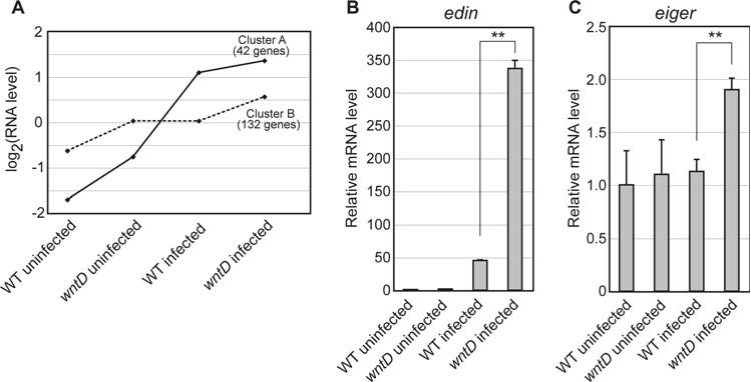
Cluster analysis identifies candidates for genes involved in increased mortality of *wntD* mutants. (A) Graph illustrating average values for genes in clusters A and B under each of the four conditions tested. Solid line indicates Cluster A, dashed line indicates Cluster B. Each data point is the mean of all three replicates of all genes in the cluster (B) Normalized Quantitative RT-PCR data for expression of *edin* under each condition. *edin* shows increased expression upon infection, and is significantly elevated in *wntD* mutants following infection. (C) Normalized Quantitative RT-PCR data for expression of *eiger* under conditions each condition. *eiger* expression is changed only in *wntD* mutants following infection. Expression levels are normalized to *Ribosomal protein 15a*, and the value of the control uninfected sample is set to 1. Error bars indicate s.e.m. Asterisks indicate significance by student t-test: ** = p<0.01.

To identify genes as candidate mediators of *wntD* mutants' infection sensitivity, cluster analysis was used [25]. Hierarchical clustering revealed several distinct groups of genes that showed correlation in their expression patterns across the four different conditions. However, two related clusters of genes were selected for further analysis based on the following rationale: the expression of genes actively contributing to pathogenesis will most likely be elevated following infection, and genes within this group that might be implicated in the more rapid lethality seen in *wntD* mutants would be expressed higher in these mutants. The average expression level under each condition for the two selected clusters (Clusters A and B) are shown in Figure 2. The clusters differ in that Cluster A shows a greater overall change in response to infection than does Cluster B (Figure 2).

Cluster A includes a number of known targets of infection, including several AMPs (Table S1). While it is certainly possible that several of these are contributing to pathogenesis in the fly, one uncharacterized gene in particular stood out based on its levels of expression. Confirmed by quantitative RT-PCR, *edin* shows strong induction by *Listeria* infection (∼45 fold), and dramatically higher levels of expression in infected *wntD* mutants versus infected controls (∼7.5 fold) (Figure 2B). Furthermore, only a 1.7 fold difference was seen between mutants and controls prior to infection, illustrating synergy between *Listeria* infection and the absence of *wntD* function on the regulation of *edin*.

Cluster B is composed of genes that show less dramatic changes in response to infection, but are still elevated in *wntD* mutants versus controls (Figure 2A, Table S2). It seems likely that this set of genes would include those that are regulated by processes aside from those sensing acute infection (Toll, Imd), and may include both mediators and markers of pathogenesis. Interestingly, this cluster includes the gene *eiger*, a TNF homolog known to mediate disease processes following *Salmonella and Mycobacterium* infections [14],[20]. In this case, using quantitative RT-PCR, we see a statistically significant elevation of *eiger* expression only in infected *wntD* mutants (Figure 2C).

### *Edin* encodes a novel protein that is misregulated in *wntD* mutants

**Figure 3:**
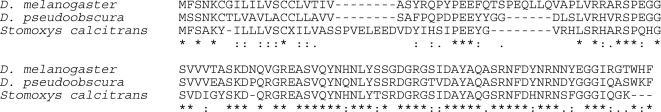
Sequence alignment of Edin with identified homologs. Alignment of three insect homologs identified by BLAST search: *Drosophila melanogaster edin*, *Drosophila pseudoobscura* GA16743-PA, and *Stomoxys calcitrans* (stable fly) EST (NCBI accession DN952940).

The *edin* gene is predicted to encode a secreted protein 115 amino acids in length (http://flybase.bio.indiana.edu/.bin/fbidq.htmlFBgn0052185). The gene has homologs in other insects, but not in other Phyla. (Figure 3). Furthermore, no known conserved domains were identified in Edin or its putative ortholog in *Drosophila pseudoobscura* and secondary structure prediction failed to identify any similar proteins or motifs based folding patterns (data not shown).

**Figure 4:**
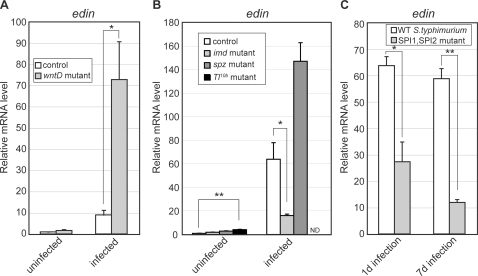
*Edin* expression is partly regulated by the Imd pathway, and is correlated with increased *S. typhimurium* pathogenesis. (A) Quantitative RT-PCR data for expression of *edin* in *yw*; *wntD* and *yw* control flies prior to and following infection with *M. luteus*. Expression is induced by infection with *M. luteus*, and expression is significantly elevated in *wntD* mutants following infection. (B) Quantitative RT-PCR data for expression of *edin* following infection with a mixture of gram-positive and gram-negative bacteria in various host genetic backgrounds. Induction is mitigated in *imd* ^10191^ mutants, demonstrating input from the Imd pathway in controlling the expression of *edin*. Flies of the genotype *spz* ^rm7^/*spz* ^2^ express *edin* at higher levels than controls. Uninfected *Tl* ^10b^/+ flies show mild induction of *edin* in the absence of infection (4.2 fold) (ND = this genotype was not assayed following infection). (C) Quantitative RT-PCR data for expression of *edin* in wild-type flies following infection with a wild-type strain of *Salmonella typhimurium* (SL1344) or a strain mutant for *SPI1* and *SPI2* (BJ66/P3F4). Values are relative to those in uninjected wild-type flies. Expression levels in all cases are normalized to *Ribosomal protein 15a*. Error bars indicate s.e.m. Asterisks indicate significance by student t-test: * = p<0.05, ** = p<0.01.

To answer the question of whether *edin* misregulation in *wntD* mutants is specific to infection with *Listeria*, *wntD* and control flies were injected with the non-pathogenic gram-positive bacteria *Micrococcus luteus*. Analysis of *Edin* expression levels prior to and following infection were monitored using quantitative RT-PCR (Figure 4A). The results are strikingly similar to those seen for *Listeria* infection; expression of *edin* is elevated 1.7-fold in *wntD* mutants compared to controls prior to infection, and 8-fold following infection. Again, a synergistic relationship is seen between infection and the presence of the *wntD* mutation. The smaller magnitude of *edin* induction seen in response to *M. luteus* compared to *Listeria* (∼10 fold versus ∼45 fold in wild-type flies) may be explained by the shorter timecourse of infection (5 hours versus 24 hours), a smaller bacterial load at the time of assay, or intrinsic differences between the two species of bacteria.

The strong regulation of *edin* in response to bacterial challenge raises the question of whether its transcription is regulated by the Toll and/or Imd pathways. To investigate this possibility, the induction of *edin* was monitored in genetic backgrounds each containing a loss of function mutation for a component in one of the pathways (Figure 4B). Mutations in *imd* reduced the expression of *edin* following infection to approximately 25% of that seen in wild type. This indicates that the Imd pathway participates in *edin* regulation, but is not strictly required for its induction following infection. By contrast, loss of function mutations in the Toll ligand *spatzle* did not reduce the transcriptional induction of *edin*, and in fact resulted in higher than normal levels of expression. This has been seen for other genes (such as *diptericin*) that do not have a strong requirement for Toll signaling, and could be due to increased survival of the bacteria in these mutants (data not shown; [4]). Levels of *edin* were slightly elevated (4-fold) in naïve flies carrying a dominantly activated allele of Toll in the absence of infection (*Toll* ^10b^; Figure 4B). These data indicate that Toll signaling may be sufficient to induce low levels of *edin* expression, but is not required for its expression.

### *Edin* is required to fight *Listeria* infections

**Figure 5:**
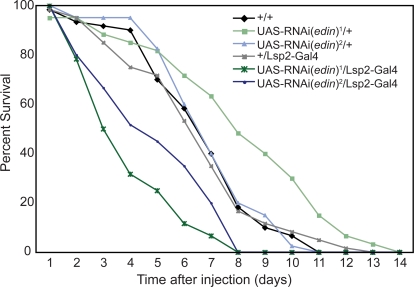
Knockdown of *edin* expression sensitizes flies to *Listeria* infection. Survival curves shown for two independent UAS-RNAi lines against *edin* controlled by the fat body driver Lsp2-Gal4. All heterozygous controls were created by mating to w^1118^, and +/+ denotes w^1118^. *Edin* knockdowns are significantly different from all three controls by Log Rank test (p<0.001). Significant differences between Listeria challenged *edin* knockdown and control flies were seen in two additional repetitions of this experiment. All experiments tested 60 flies per condition.

In order to investigate whether Edin plays an essential role in disease progression, we knocked down its expression using two independently made UAS-driven RNA interference (RNAi) constructs. *Edin* expression was knocked down using the fat body driver Lsp2-Gal4 to ablate its activity in a major immune tissue. Edin knockdown flies displayed increased sensitivity to *Listeria monocytogenes*, with flies dying significantly faster than all controls (p<0.001) (Figure 5). This demonstrates that *edin* is required for an effective host response against *Listeria* infection. Interestingly, bacterial loads in *edin* knockdown flies were not significantly different from controls (data not shown). This places *edin* among several previously identified genes that affect a fly's endurance during *Listeria* infection rather than its ability to combat bacterial growth [26]. While the mechanism for this effect is unknown, we hypothesize that knockdown of *edin* expression alters the physiology of the fly in a way that makes it more susceptible to *Listeria* pathogenesis.

### Detrimental effects of Edin misregulation

Immunity can be a double-edged sword that has to be regulated precisely to help defend against infection while limiting damage to the body. Overexpression of genes misregulated during an immune response led us to *edin* and we found that it is required for fly survival during an *L.monocytogenes* infection. Next, we thought it was of great interest to determine whether Edin expression contributed to pathology. We first looked for more evidence that Edin was associated with pathology under different circumstances. We compared the expression of *edin* following infection of wild-type flies with wild-type *Salmonella typhimurium* or a *SPI1*, *SPI2* mutant strain of *Salmonella* that has decreased pathogenicity [14]. As shown in Figure 4C, *edin* was expressed at significantly higher levels during the course of a wild-type *Salmonella* infection compared to the less pathogenic strain at both time points tested. The more dramatic difference was seen later in infection, when *edin* transcript levels were over 5-fold higher in flies infected with wild-type *Salmonella* (Figure 4C). These data add more correlative evidence that Edin is associated with pathogenesis.

**Figure 6:**
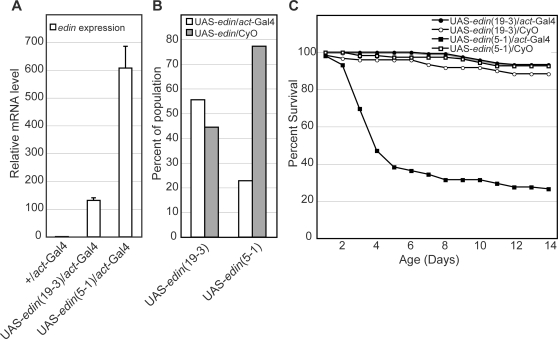
Overexpression of *edin* negatively impacts survival. (A) Quantitative RT-PCR data for RNA levels of *edin* following overexpression with the *actin*-Gal4 system. Two different insertions of UAS-*edin* were tested, with one (5-1) showing 5-fold higher levels of expression than the other (19-3). (B) Proportion of progeny carrying *actin*-Gal4 versus the CyO balancer in crosses between *actin*-Gal4/CyO and each insertion of UAS-*edin*. Viability is decreased in *actin*-Gal4/UAS-*edin*(5-1), leading to lower representation of this genotype within the progeny of that cross (n = 1354 for insertion 19-3 cross and 865 for insertion 5-1 cross). (C) Survival of the 4 populations represented in part b, over the 2 weeks following eclosion. *actin*-Gal4/UAS-*edin*(5-1) flies exhibit a marked decrease in survival in the first four days after eclosion. Between 102 and 120 flies were measured for each genotype.

Do *edin* expression levels affect survival? To answer this question, we first overexpressed *edin* using the UAS/Gal4 system. Two different insertions of the p-element carrying UAS-*edin* resulted in varied levels of expression, with one insert (19-3) causing expression levels ∼100 fold over wild type when combined with *actin*-Gal4, and the other overexpressing *edin* over 500 fold (Figure 6A). We observed that the higher level of expression resulted in significant levels of lethality prior to and following eclosion (Figure 6B,C). Flies strongly overexpressing *edin* survived to adulthood at a frequency less than 50% of expected, compared to 111% for the lower expresser. The value greater than 100% can most likely be attributed to non-specific deleterious effects of carrying the CyO balancer. The average lifespan of those flies surviving to adulthood was also significantly reduced in the context of strong overexpression of *edin* (Figure 6C). Given that *wntD* mutants infected with *L. monocytogenes* displayed similar levels of expression to the strong insertion of UAS-*edin* (about 350 fold over uninfected wild-type flies; Figure 2B), it is possible that *edin* expression is contributing to the rapid mortality of these mutants.

Taken together with the observation that *edin* loss of function mutants show increased sensitivity to *L.monocytogenes*, these data support a model in which *edin* expression must be tightly controlled during a host response to infection: moderate induction is essential to an effective response, but uncontrolled, high levels of expression become detrimental to the host animal.

## Discussion

The idea that an elevated immune response could be detrimental to an infected host is at first unintuitive. However, it is well established that, like most other biological processes, proper regulation and containment of the immune response is critical to an animal's viability. In mammals, LPS-triggered TNF release at a site of injury/infection is critical to mobilize the immune and inflammatory processes required to fight the infection, but in the rare cases when this reaction becomes uncontrolled and systemic, the shock will rapidly kill the host [17]. Studies in the fly have shown that genetic removal of a TNF-like molecule called Eiger increases flies' longevity during some infections, but decreases it during others [14],[20]. Thus eiger appears to be a double-edged sword – necessary for fighting some infections, but not without a cost to the host. Similarly, flies carrying *Tl* ^10b^ mutations, which dominantly activate the Toll pathway, die more rapidly from *Drosophila* X virus infection, despite lower viral loads [27],[28], and over-activation of the IMD pathway has a negative impact on larval survival during bacterial infection [28]. These results imply that both the Toll and IMD pathways activate the transcription of genes that have a deleterious effect on a fly's survival during pathogenic infection, one of which could well be *eiger*. In light of these findings, the observation that *wntD* mutants die more quickly from *Listeria* infection, while hyperactivating immune genes, is less surprising. Furthermore, this phenotype is suppressed by loss of *dorsal*, implying that Dorsal is actively regulating processes that decrease the fly's survival [21].

### *Edin* as a candidate mediator of pathogenesis

We presented two experiments that compared the expression profiles of flies undergoing two different levels of pathogenesis: *wntD* versus control flies following *L. monocytogenes* infection, and wild-type *S. typhimurium* versus a SPI1, SPI2 mutant strain. In both cases the gene *edin* was strongly elevated in the flies closer to death. In comparing *wntD* mutant versus control flies following *Listeria* infection, RNA samples were taken 1 day after infection, shortly before the mutants exhibit a sharp decrease is survival [21]. Expression of *edin* was about 8-fold higher in the *wntD* mutants. Similarly, at 7 days post *Salmonella* infection, flies infected with wild type have begun to die, while those infected with a *SPI1*,*SPI2* mutant strain will live for several more days despite carrying dramatically higher loads of bacteria [14]. In this case, we observed a 5-fold elevation in *edin* expression in the flies beginning to die. Thus, high *edin* expression is correlated with increased pathogenesis, although a causal relationship is not established by these data.

Two results strongly suggest that *edin* induction is not downstream of pathogenesis. First, *edin* expression is elevated following infection with *M. luteus*, a non-pathogenic bacterium, and is more strongly induced in *wntD* mutants (Figure 2A). These data demonstrate that pathogenesis is not required for *edin* expression. Second, the Imd pathway appears to play a significant role in regulating *edin*, and this pathway is acutely induced upon recognition of bacterial moieties and does not strictly depend on pathogenesis [29]–[31].

Could Edin play a causal role upstream of pathogenesis? The induction of *edin* during *M. luteus* infection without any demonstrable pathogenesis suggests that the amount of Edin produced during this infection is not sufficient to elicit pathogenesis. However, these levels are approximately 5-fold lower than those seen for *Listeria* infection and persist for less than a day (data not shown), in contrast to the chronic induction during infection with *Listeria* or *Salmonella*. Furthermore, the lethality induced by strong chronic overexpression of *edin* using the UAS/Gal4 system implies that this gene can induce processes detrimental to a fly's survival that could be affecting viability during persistent infections. Though Edin can be shown to cause pathology when overexpressed, it is difficult to produce clean evidence that this occurs during infection, because the overexpression of many genes can cause pathology; therefore it remains a suggestion.

### Is Edin an AMP?

*Edin* shows several characteristics consistent with it being an AMP. First, it is strongly induced by infection; *edin* was the second most highly induced gene in wild-type flies following *L. monocytogenes* infection, and the most highly induced gene in *wntD* mutants. Second, *edin* is predicted to encode a short peptide and a processed form has been observed circulating in the hemolymph of infected flies [23]. However, *edin* also displays properties that would make it unique among AMPs, suggesting that it may be more broadly affecting physiology, perhaps in a cytokine-like role similar to that of *eiger*. For instance, the expression of this gene is required for normal survival following *L. monocytogenes* infection. While necessity for the signaling pathways controlling AMP expression is well documented, this is the first case of an individual putative AMP being necessary to fight infections {Ferrandon, 2007 #329}. This requirement during infection, combined with the toxicity observed upon overexpression suggests that Edin may be a powerful component of the immune response that must be tightly regulated to optimize survival. Further analysis of *edin* and other genes that are differentially regulated during pathogenesis could provide interesting clues into the complicated and evolving nature of the host-pathogen interaction.

## Materials and Methods

### *Drosophila* strains

The construction of *wntD* mutants was described previously [21]. Any reference to *wntD* mutant is the genotype *yw*; *wntD* ^KO1^. References to ‘wild type’ refer to *yw*; +/+; +/+ or *w* ^1118^; +/+; +/+ if so noted. pP[UAS-*edin*] was constructed by amplifying the *edin* open reading frame using PCR, and cloning this fragment into the Xba-1 site of pPUAST [32]. UAS-RNAi(edin)^2^ was created at the VDRC (transformant 14289). UAS-RNAi(edin)^1^ was generated by PCR amplification of the complete cDNA with XbaI sites at both 5′ and 3′ ends. This fragment was subcloned into the pWIZ vector [33] in two sequential cloning steps on either side of a small intron in a 3′to 5′/5′to 3′ orientation. Expression of the double-stranded RNA is under the control of the UAS promoter and is transformed into a snapback hairpin upon splicing of the small intron. Flies carrying expression constructs were created using standard p-element transformation techniques.

### Bacterial injections

All injections were done using male flies aged one week post eclosion. A culture of *Listeria monocytogenes* was diluted to an OD(600) of 0.1, and a 25 nL volume was injected abdominally using a pulled glass needle as previously described [15]. Groups of 20 flies of each genotype were injected in an alternating manner to control for variability over time. Flies were maintained on non-yeasted, standard dextrose medium at 25°C, 65% relative humidity, and survival was monitored daily. *Micrococcus luteus* and *Salmonella typhimurium* was injected as described for *L. monocytogenes*. For experiments on the regulation of *edin*, flies of different genetic backgrounds were injected with a mixture of *M. luteus*, *L. monocytogenes*, and *E. coli*, each at an OD(600) of 0.1.

### Quantitative RT-PCR

Groups of 6 flies were collected, crushed in 150 µl of Trizol reagent, and RNA was extracted according to the manufacturer's recommendations. 1 µl RNA was used for subsequent reverse transcription using the ThermoScript RT-PCR system (Gibco BRL), following the manufacturer's instructions and using a random hexamer as primer. Quantitative PCR was preformed in a LightCycler (Roche), using the LightCycler FastStart DNA Master^PLUS^ SYBR green I kit (Roche) and following the manufacturer's recommendations.

Primers used for PCR were as follows:

*edin*: TCCAGTGGCACCCTTGGTA and TAGTTGTTCCGATTGTAGTCGAA
*eiger*: GATGGTCTGGATTCCATTGC and TAGTCTGCGCCAACATCATC
*ribosomal protein 15a*: TGGACCACGAGGAGGCTAGG and GTTGGTGCATGGTCGGTGA

### Gene expression profiling

Groups of 30 *yw;wntD* ^KO1^ or *yw* flies (some previously infected with *Listeria monocytogenes* as described above) were collected in 1.5 mL microfuge tubes. Each experiment was done in triplicate, for 12 total samples. Conditions were: *yw* uninjected, *yw;wntD* ^KO1^ uninjected, *yw* 24 hours post *Listeria* infection, *yw;wntD* ^KO1^ 24 hours post *Listeria* infection. Flies were crushed in 1 mL Trizol reagent, and RNA was isolated using the manufacturer's recommendations. 15 µg of each RNA sample was then used for cDNA synthesis, which was done using the one cycle cDNA synthesis (Affymetrix) and following the manufacturer's recommendations. cRNA was also synthesized using the manufacturer's protocol, and 20 ug was used for the subsequent fragmentation step. cRNA was hybridized to Affymetrix Drosophila Genome 2.0 arrays by the Stanford Protein and Nucleic Acid Biotechnology Facility (http://pan.stanford.edu). Arrays were analyzed using the Affymetrix GCOS software to produce normalized values for each probe set on each array.

### Clustering

Clustering was performed on a dataset in which genes were included only if they were marked as “present” by GCOS in all 3 samples of at least one condition. Clustering was done using Cluster 3.0 for Mac OS X (http://bonsai.ims.u-tokyo.ac.jp/mdehoon/software/cluster/software.htm). Parameters used for clustering were: Data was log transformed and genes were centered. Data was filtered to include only genes where the difference between the highest and lowest values was greater than or equal to 1 (representing a two-fold change or greater). Hierarchical clustering was performed using the centroid linkage algorithm. Clusters were viewed using Java Treeview software (http://genetics.stanford.edu/alok/TreeView/). Gene identities and annotations shown in Tables S1 and S2 were retrieved using the Netaffx analysis webpage (http://www.affymetrix.com/analysis/index.affx).

## Supporting Information

Table S1 Genes in cluster A
(0.11 MB DOC)

Table S2 Genes in cluster B
(0.21 MB DOC)
