# Supplementary material for: Pathogenesis of Listeria-Infected Drosophila wntD Mutants Is Associated with Elevated Levels of the Novel Immunity Gene edin
Source: PLoS Pathog. 2008 Jul 25;4(7):e1000111. doi: 10.1371/journal.ppat.1000111 (PMC2453329; doi:10.1371/journal.ppat.1000111)
Supplement: Table S1 — Genes in cluster A (0.11 MB DOC) [file ppat.1000111.s001.doc]

Table S1

| **Genes in Cluster A** | | |
| --- | --- | --- |
| **Probe Set ID** | **Gene Title** | **Gene Symbol** |
| 1623565_at | --- | CG17278 |
| 1623643_s_at | Imaginal disc growth factor 3 | Idgf3 |
| 1623716_at | Actin-related protein 66B | Arp66B |
| 1623903_at | --- | CG18473 |
| 1624392_s_at | Malvolio | Mvl |
| 1625124_at | Attacin-A | AttA |
| 1625164_at | Turandot X | TotX |
| 1626319_a_at | Immune induced molecule 10 | IM10 |
| 1627246_at | --- | Pcaf |
| 1627551_s_at | Attacin-B /// Attacin-A | AttB /// AttA |
| 1627613_at | Metchnikowin | Mtk |
| 1627759_at | --- | CG30080 |
| 1627962_at | --- | CG31749 |
| 1628229_at | Serpin-27A | Spn27A |
| 1629072_at | suppressor of white-apricot | su(w^a^) |
| 1629530_at | Immune induced molecule 23 | IM23 |
| 1629566_at | --- | CG8834 |
| 1629791_at | --- | CG17271 |
| 1630163_at | --- | CG32373 |
| 1631475_at | Attacin-D | AttD |
| 1631486_at | --- | p38c |
| 1632719_at | Cecropin C | CecC |
| 1633053_at | Immune induced molecule 1 | IM1 |
| 1633145_at | Peptidoglycan recognition protein LF | PGRP-LF |
| 1633224_at | --- | CG9631 |
| 1633237_at | Imaginal disc growth factor 1 | Idgf1 |
| 1633400_at | cueball | cue |
| 1633488_at | astray | aay |
| 1633545_at | --- | PGRP-SD |
| 1634271_at | Defensin | Def |
| 1634366_at | --- | EDIN |
| 1634733_at | --- | CG3831 |
| 1635189_at | Drosomycin | Drs |
| 1635507_at | --- | CG4269 |
| 1636257_at | --- | CG10688 |
| 1636410_at | --- | CG3505 |
| 1636639_at | Signal peptide protease | Spp |
| 1637577_at | Zinc/iron regulated transporter-related protein 3 | Zip3 |
| 1638235_at | Diptericin B | DptB |
| 1639019_s_at | Immune induced molecule 10 | IM10 |
| 1640855_at | --- | CG11864 |
| 1641419_at | Attacin-C | AttC |
