# Supplementary material for: Pathogenesis of Listeria-Infected Drosophila wntD Mutants Is Associated with Elevated Levels of the Novel Immunity Gene edin
Source: PLoS Pathog. 2008 Jul 25;4(7):e1000111. doi: 10.1371/journal.ppat.1000111 (PMC2453329; doi:10.1371/journal.ppat.1000111)
Supplement: Table S2 — Genes in cluster B (0.21 MB DOC) [file ppat.1000111.s002.doc]

Table S2

|  | **Genes in Cluster B** |  |
| --- | --- | --- |
| **Probe Set ID** | **Gene Title** | **Gene Symbol** |
| 1623416_at | --- | CG33265 |
| 1623813_at | --- | CG33307 |
| 1624054_at | Adenosine deaminase-related growth factor A | Adgf-A |
| 1624137_at | --- | CG11911 |
| 1624272_at | --- | CG10618 |
| 1624328_a_at | Septin-1 | Sep-1 |
| 1624354_at | non-stop | not |
| 1624415_s_at | --- | CG8121 |
| 1624568_at | --- | CG7985 |
| 1624817_at | --- | CG3212 |
| 1625033_at | --- | CG16791 |
| 1625245_at | --- | CG3759 |
| 1625250_at | --- | CG5802 |
| 1625405_at | yellow-c | yellow-c |
| 1625436_at | Urate oxidase | Uro |
| 1625512_s_at | --- | --- |
| 1625542_a_at | Tropomyosin 1 | Tm1 |
| 1625997_s_at | --- | --- |
| 1626196_at | --- | CaBP1 |
| 1626233_at | --- | CG8965 |
| 1626933_s_at | --- | CG2469 |
| 1627184_at | --- | scaf6 |
| 1627327_at | drosomycin-5 | dro5 |
| 1627552_at | --- | --- |
| 1627620_at | --- | CG14034 |
| 1627872_at | --- | CG3770 |
| 1627890_at | Glutathione S transferase D10 | GstD10 |
| 1627961_a_at | --- | CG18507 |
| 1628187_s_at | --- | CG9691 |
| 1628578_at | --- | CG10651 |
| 1629050_at | --- | CG10477 |
| 1629182_at | --- | CG10140 |
| 1629308_at | --- | CG17192 |
| 1629442_at | eiger | egr |
| 1629715_at | --- | CG7891 |
| 1629799_at | --- | CG32382 |
| 1629938_at | Copper transporter 1A | Ctr1A |
| 1629969_at | --- | CG11426 |
| 1630088_at | --- | CG16743 |
| 1630109_at | --- | CG8628 |
| 1630159_at | --- | CG11672 |
| 1630498_at | --- | --- |
| 1630699_at | &ngr; integrin | Int-&ngr; |
| 1630831_at | --- | CG7695 |
| 1630915_at | --- | --- |
| 1631062_at | --- | CG2023 |
| 1631121_at | --- | CG7267 |
| 1631323_a_at | --- | CG2663 |
| 1631571_a_at | spire | spir |
| 1631660_at | --- | CG15065 |
| 1631670_at | Serine protease inhibitor 2 | Spn2 |
| 1631693_at | --- | CG9616 |
| 1631715_at | --- | CG14872 |
| 1632168_at | male-specific opa containing gene | msopa |
| 1632212_at | --- | CG14401 |
| 1632415_s_at | --- | CG6169 |
| 1632639_at | --- | CG13941 |
| 1632648_at | --- | --- |
| 1632834_a_at | --- | COP |
| 1632850_at | --- | CG7338 |
| 1633002_at | --- | CG2196 |
| 1633031_at | --- | CG8299 |
| 1633094_a_at | twin of eyeless | toy |
| 1633378_at | Galactose-specific C-type lectin | Lectin-galC1 |
| 1633425_at | --- | CG2191 |
| 1633483_a_at | --- | CG14207 |
| 1633512_at | twin of eyeless | toy |
| 1633523_at | Porphobilinogen synthase | Pbgs |
| 1633592_a_at | Cellular Repressor of E1A-stimulated Genes | CREG |
| 1633812_at | --- | CG9080 |
| 1634144_at | --- | CG12958 |
| 1634164_at | Syntaxin Interacting Protein 1 | SIP1 |
| 1634219_a_at | thioredoxin peroxidase 1 | Jafrac1 |
| 1634318_at | --- | CG33110 |
| 1634525_at | --- | CG7738 |
| 1634633_s_at | --- | --- |
| 1634731_at | --- | Cyp4p3 |
| 1634742_at | Sorbitol dehydrogenase-2 | Sodh-2 |
| 1634773_at | Signal sequence receptor | SsR |
| 1634834_at | --- | CG4053 |
| 1635109_at | --- | CG5888 |
| 1635294_at | --- | CG9312 |
| 1635512_at | --- | CG11893 |
| 1635537_at | anti-silencing factor 1 | asf1 |
| 1635688_at | --- | CG15210 |
| 1635793_at | --- | CG11413 |
| 1635878_s_at | --- | CG17571 |
| 1636330_at | --- | CG17107 |
| 1636567_at | --- | CG31199 |
| 1636759_at | --- | CG8303 |
| 1636784_at | Serine protease inhibitor 3 | Spn3 |
| 1637086_at | --- | CG11843 |
| 1637224_at | --- | CG13905 |
| 1637275_a_at | --- | CG13335 |
| 1637350_at | --- | CG31086 |
| 1637367_at | --- | CG10799 |
| 1637388_at | --- | CG13511 |
| 1637492_at | Trypsin | Try |
| 1637557_a_at | --- | CG10724 |
| 1637660_at | --- | CG5150 |
| 1637738_at | --- | CG5417 |
| 1637926_s_at | --- | CG14084 |
| 1637963_at | --- | CG14191 |
| 1638021_at | --- | CG4757 |
| 1638176_at | Saccharomyces cerevisiae UAS construct a of Beller | Magi |
| 1638265_s_at | --- | --- |
| 1638272_at | Signal recognition particle protein 19 | Srp19 |
| 1638528_at | --- | CG15531 |
| 1638722_at | --- | CG16807 |
| 1638783_at | mitochondrial ribosomal protein L48 | mRpL48 |
| 1638845_at | --- | CG18643 |
| 1638890_at | Translocon-associated protein | Tap |
| 1639452_at | --- | CG14984 |
| 1639457_at | --- | CG32284 |
| 1639503_at | Tubulin at 60D | Tub60D |
| 1639528_at | --- | CG3252 |
| 1639643_at | --- | CG18557 |
| 1639671_at | eiger | egr |
| 1639694_s_at |  | CG10102 |
| 1639784_at | Saccharomyces cerevisiae UAS construct a of Cheng | Pros45 |
| 1639810_at | Dead box protein 73D | Dbp73D |
| 1640155_at | --- | --- |
| 1640303_a_at | pastrel | pst /// Dyakpst |
| 1640405_at | --- | l(2)01810 |
| 1640540_at | --- | CG30273 |
| 1640603_at | --- | CG32590 |
| 1640881_at | --- | CG16762 |
| 1641342_at | --- | cag |
| 1641570_s_at |  | CG31683 |
